# Economic valuation of a holistic rewilding approach in multifunctional landscapes: Evidence from the German Oder Delta

**DOI:** 10.1007/s13280-025-02143-7

**Published:** 2025-02-18

**Authors:** Julian R. Massenberg

**Affiliations:** 1 Department of Economics, Helmholtz Centre for Environmental Research—UFZ, Permoserstraße 15, 04318 Leipzig, Germany; 2 Department of Environmental Science, Aarhus University, Frederiksborgvej 399, 4000 Roskilde, Denmark

**Keywords:** Discrete choice experiment, Ecosystem services, Human–nature relationships, Rewilding, Value pluralism, Willingness to pay

## Abstract

The academic discourse on rewilding has primarily focused on its ecological dimensions, yet rewilding initiatives also have the potential to combine ecological restoration with regional economic development and societal well-being. Using a discrete choice experiment, this study investigates public preferences and willingness to pay for rewilded landscapes in the Oder Delta and the underlying motivations that influence these preferences. The findings show a general willingness to support a holistic rewilding approach among the local population of Mecklenburg–West Pomerania. However, the results also highlight heterogeneous preferences with respect to different aspects of rewilding as well as underlying motivations. By providing insights into local preferences for rewilded landscapes, this research contributes to informing local rewilding initiatives and overarching conservation policies. It emphasises the importance of navigating trade-offs and conflicts inherent in rewilding projects and land use while emphasising the need for further research on the determinants of preferences and willingness to pay.

## Introduction

In recent years, rewilding has gained increasing attention, among others, as a restoration approach which emphasises the importance to restore natural processes while reducing human interventions. Thereby, the overarching ecological aim is to promote biodiversity, provision of ecosystem services, and resilience to climate change on a landscape level. However, rewilding is a fuzzy concept with different meanings, i.a., with focus on restoration of wildlife and species reintroduction, productive land abandonment or restoration of self-sustaining ecosystems (see Jørgensen 2015; Massenberg et al. 2023b for a detailed discussion). Accordingly, the few economic studies that have so far investigated rewilding as a concept referred to different conceptions of it. For example, van Berkel and Verburg (2014) investigated public preferences for rewilding understood in terms of agricultural abandonment, Granado-Díaz et al. (2022) analysed famers’ stated preferences for agricultural abandonment, Martínez-Jauregui et al. (2020) analysed stated preferences for rewilding understood in terms of wildlife populations, Schou et al. (2020) carried out a (private) cost–benefit analysis of establishing nature reserves for rewilding to compare the cost-effectiveness relative to agri-environmental schemes, Williams et al. (2022) applied value transfer in the context of marine ecosystems (kelp forest restoration), and most recently Dunn-Capper et al. (2024) studied stated preferences for the ecological concept of rewilding developed by Perino et al. (2019) which focuses on restoration of key natural processes and self-sustaining ecosystems.

**Fig. 1:**
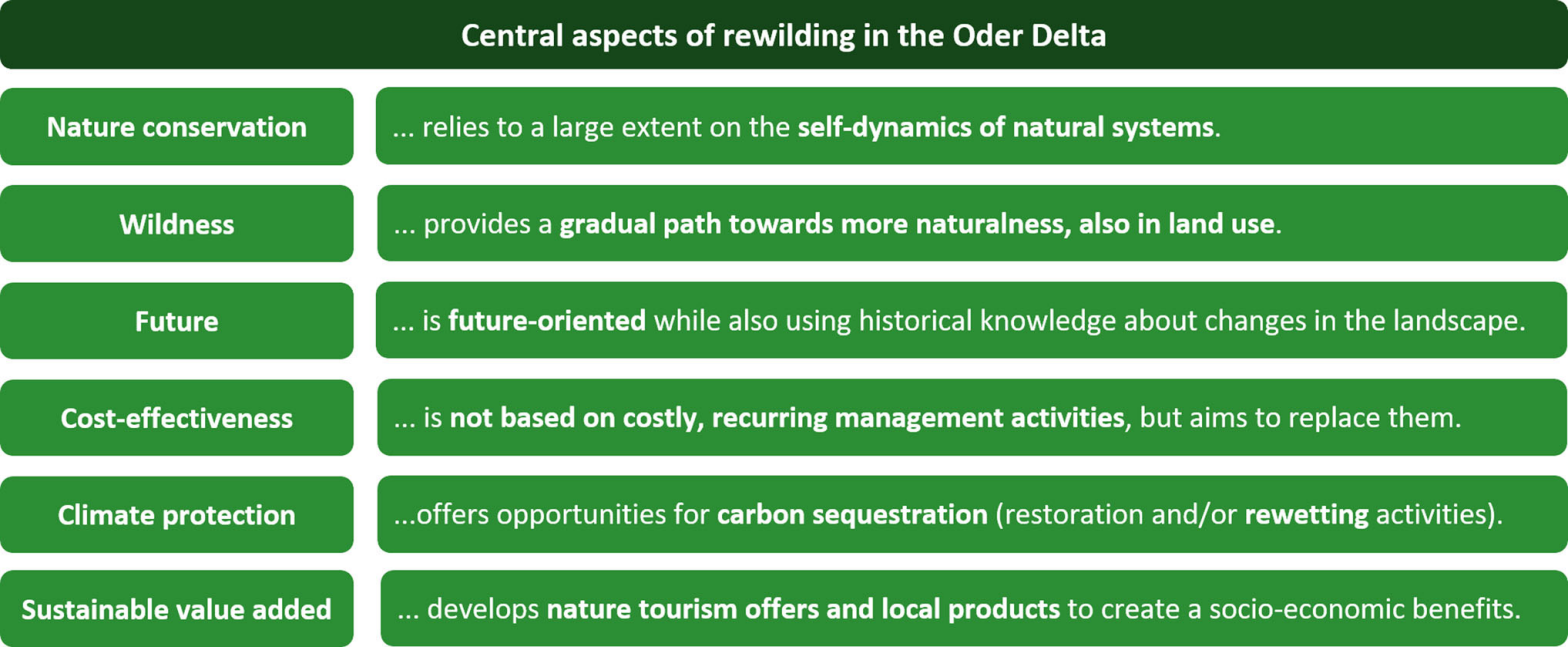
Central aspects of rewilding in the Oder Delta as described by the transdisciplinary project partner (ROD). Source: ROD (2022, p. 4)

In contrast to the academic debate with its primarily ecological focus, rewilding initiatives have emerged as a compelling narrative that integrates ecosystem restoration and nature conservation with regional economic development and societal well-being (Jepson et al. 2018; Jepson 2019). Therefore, in this study, the term holistic rewilding refers to an expanded interpretation that builds upon the ecological foundation of restoring ecosystems self-sustainability (Perino et al. 2019) but also integrates social, economic, and cultural dimensions. This broader approach emphasises the restoration of wildness, understood as the self-regulation of ecosystems and their ability to sustain themselves, while also acknowledging the role of humans as part of the socio-ecological system (see, e.g., Massenberg et al. 2023b). Thus, it aims at strengthening local ecosystems as well as local economies and development. Put differently, a holistic rewilding approach involves restoring ecosystems to a natural, self-sustaining state by integrating biodiversity restoration, ecological processes, landscape connectivity, as well as socio-economic and cultural considerations (Jepson et al. 2018; Massenberg et al. 2023b). Such a holistic approach has the potential to involve and engage local communities, to promote economic opportunities, for example through nature-based tourism, and may thereby support policies that advance both ecological restoration as well as sustainable development (Jepson et al. 2018; Massenberg et al. 2023b). In the European context, this is an important aspect of rewilding due to the long-lasting human influence and associated multifunctionality of landscapes (Drenthen 2018; Jepson et al. 2018; Massenberg et al. 2023b). Landscapes are often shaped by complex interactions among social, cultural, and ecological factors (Fischer et al. 2012), and alterations to these landscapes can influence human–nature relations and values (Riechers et al. 2020). With reference to the EU Biodiversity Strategy for 2030, the rewilding initiative in the case study area aims to actively contribute to the goals of ecosystem restoration, reduction in pesticide use, promotion of agroecological practices, and reversal of pollinator decline (ROD 2022, p. 7, see also Fig. 1).

Furthermore, rewilding projects hold potential to generate economic opportunities for local communities mainly associated with nature-based tourism through habitat restoration and increase in wildlife (see Faure et al. (2024) for a detailed discussion). In this line of thought, rewilding may contribute to regional economic development and help address social vulnerabilities. Additionally, it could facilitate a reconnection with nature through nature-based activities, education, and recreation, thereby highlighting the role of biodiversity, particularly large charismatic animals, for recreation and tourism. Furthermore, rewilding can support additional ecosystem services such as carbon sequestration (Navarro and Pereira 2012) and pollination (Garrido et al. 2019). Particularly in Germany, including the case study region, agriculture plays a significant role in the economy, and pollination is essential for agricultural productivity (Navarro and Pereira 2012; Nogué et al. 2016). Incorporating these facets and ecosystem services in land use policies and planning is crucial for recognising both benefits and trade-offs, highlighting the need to integrate economic environmental valuation in decision-making (Viglizzo et al. 2012). Against this background, also the trade-offs between ecosystem restoration, biodiversity conservation, ecosystem service provision, land use, landscape aesthetics, cultural heritage, and regional development should be central to rewilding discussions. Alongside its ecological benefits, rewilding raises several important issues that must be considered associated with the alteration of land management practices and the potential loss of income through these changes. The shift in land management practices may also impact landscape aesthetics, cultural heritage, and local livelihoods. Thus, rewilding may be seen as a threat to cultural identity in regions where traditional land use practices have shaped local communities. In such cases, the shift in land use associated with rewilding could conflict with deeply rooted cultural values (Höchtl et al. 2005; Drenthen 2018). Moreover, rewilding’s positive effects on biodiversity may be accompanied by increased natural disturbances and/or human–wildlife conflicts (Perino et al. 2019). These conflicts may require additional management interventions or compensation mechanisms (Hearn et al. 2014; Law et al. 2017; Lennox et al. 2018). Aspects which have been long recognised in the ecosystem service literature (see, e.g., Haines-Young and Potschin 2013; Scholte et al. 2015; Jacobs et al. 2016). In essence, the quality of rewilded landscapes and their appreciation by the population are key considerations in terms of acceptance and (perceived) legitimacy. Thus, as rewilding gains prominence, understanding its economic value and the societal values associated with rewilded landscapes becomes increasingly relevant (Schulte to Bühne et al. 2022; Massenberg et al. 2023b).

In this light, this study analyses public preferences for a rewilded landscape in the Oder Delta and underlying motivations behind preferences, aiming to uncover the deeper values, beliefs, and attitudes influencing the choices within the context of rewilding landscapes. Building upon existing literature and theoretical frameworks, the complexity of human–nature relations is explored, recognising the intricate interplay between ecological and socio-cultural factors (see, e.g., Breyne et al. 2021). Follow-up questions provide additional information to better understand participants' motivations. This deeper insight on the human perspectives on rewilded landscapes helps inform local rewilding initiatives as well as overarching conservation policies.

This study was conducted as part of a transdisciplinary research project, involving collaboration between experts from the social and natural sciences as well as the involvement of a local rewilding NGO. The project’s overall aim was to critically evaluate the concept of rewilding from an ecological, economic, and socio-cultural perspective. The involvement of local stakeholders ensured that the research reflected the regional context and addressed the practical realities of implementing rewilding initiatives. The objective was to conduct a scientifically grounded evaluation of rewilding’s potential benefits, challenges, and limitations within the local context, while also considering broader implications. The author’s disciplinary background in environmental and ecological economics influenced the research questions and analysis addressed by this study. However, the study aims to provide a balanced, objective evaluation of rewilding, considering both its diverse ecological as well as socio-economic and cultural dimensions. The remainder of this article is organised as follows: Firstly, the methods used in the study are described (Section “Methods”). Subsequently, the obtained empirical results together with the analysis of underlying motivations are presented (Section “Results”). Following on, results are discussed (Section “Discussion”) and lastly, conclusions are drawn (Section “Conclusions”).

## Materials and methods

### Caste study area

**Fig. 2:**
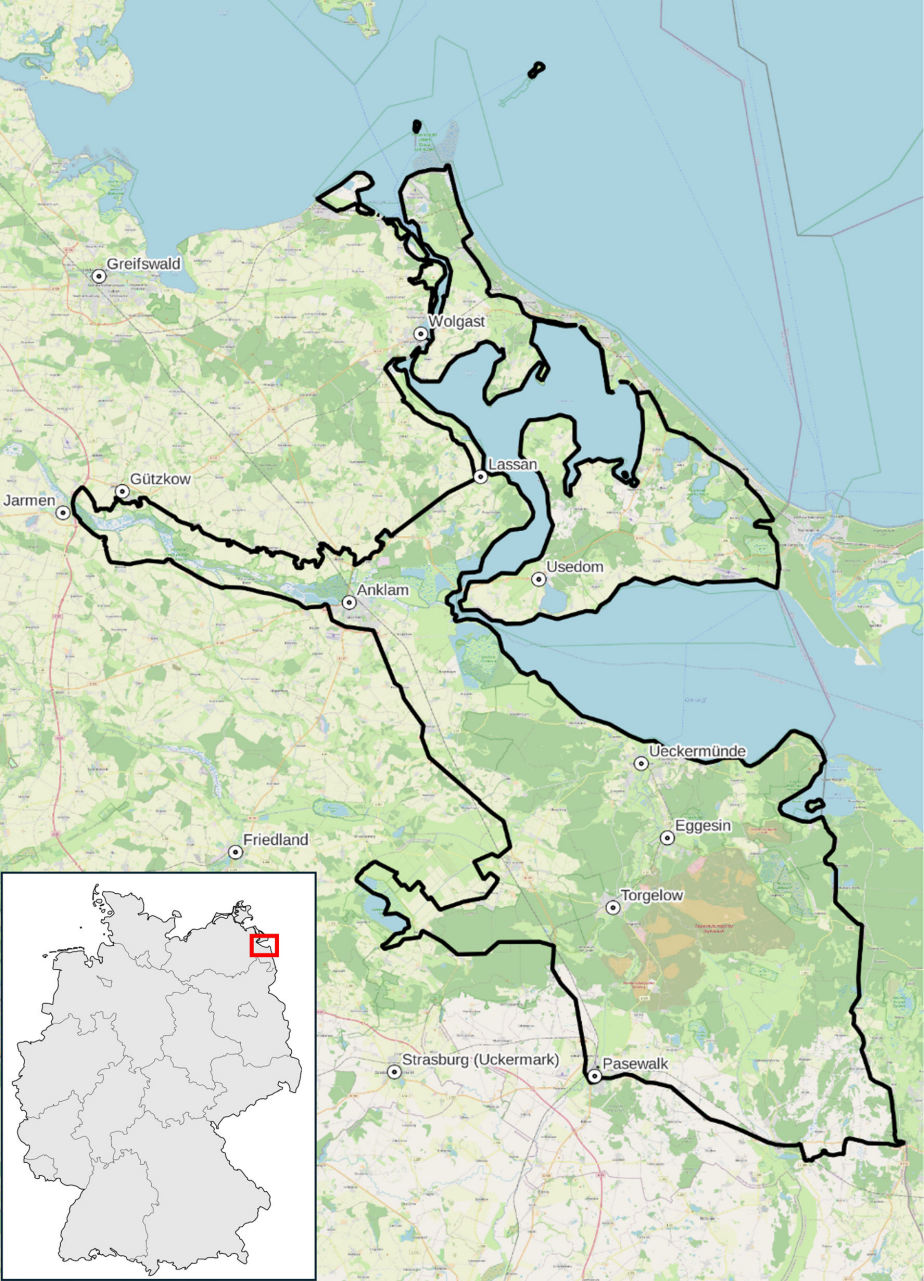
Case study area, Mecklenburg–West Pomerania, Germany

The study area is one of the ten European rewilding areas and located in the state of Mecklenburg–West Pomerania, more specifically in the German part surrounding the Szczecin Lagoon, hereinafter referred to as the ‘Oder Delta’. Figure 2 depicts the study area covering an area of approximately 1500 km^2^ (excluding water surfaces). The Oder Delta is an ecologically significant region characterised by a diverse mosaic of near-natural terrestrial, semi-aquatic, aquatic, and marine ecosystems. The area is home to a range of species, some of which are projected to return, including the white-tailed eagle, European bison, European beaver, moose, wolf, Baltic sturgeon, and grey seal. Besides their ecological impact, these species may offer the potential for generating local economic benefits, particularly through tourism, and foster socio-cultural appreciation for nature. Given these ecological and socio-economic opportunities, the Oder Delta was recognised as the eighth model area by the non-profit organisation "Rewilding Europe" in 2015. This recognition has led to the implementation of various rewilding activities aimed at improving the ecological status of natural habitats as well as generating ecosystem services. These efforts aim not only at contributing to biodiversity conservation but also at supporting to revitalise the region, which is facing population decline, by providing new economic opportunities that synthesise rewilding efforts and local development. At the forefront of these efforts is the association "Rewilding Oder Delta" e.V. (ROD), founded in 2019 to coordinate and promote rewilding activities across the region. ROD works closely with partners in nature conservation, tourism, and public authorities, in order to ensure that the rewilding initiatives are considering local economic and socio-cultural development. ROD served as the primary regional project partner in this study, facilitating stakeholder engagement and providing on-the-ground knowledge to enhance the practical relevance of the research.

**Fig. 3:**
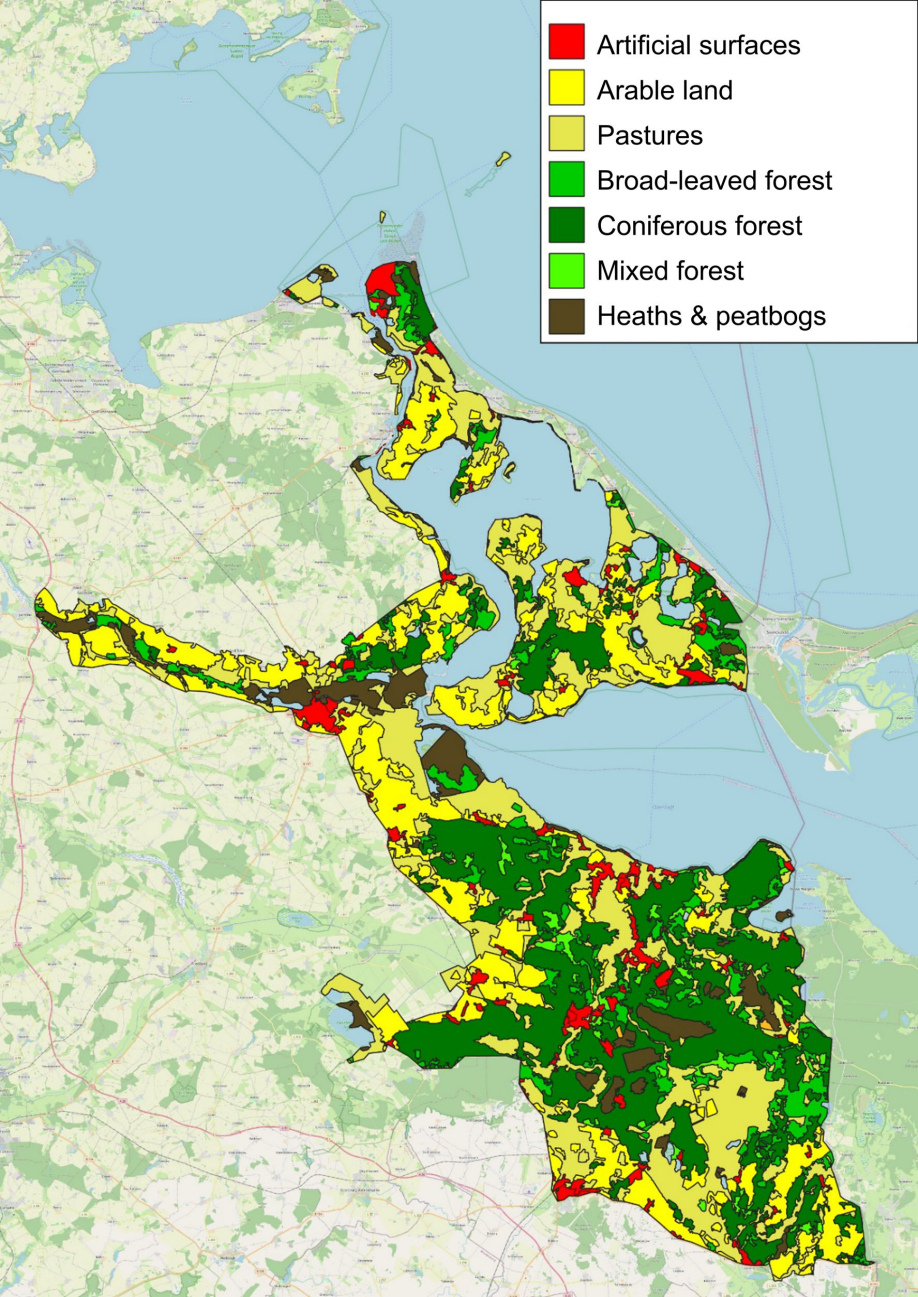
Overview of corine land cover in the caste study area

Figure 3 illustrates the current landscape composition based on the Corine Land Cover. The region features a diverse mosaic of expansive forests, wetlands, moors, dry heath landscapes, rivers, lakes, and agricultural areas. This landscape mosaic, particularly the protected natural habitats, is populated by a diversity of animals and plants. At present, roughly half of the area is used for agriculture (approximately 23% arable fields and 31% pastures). More than one-third of the area consists of forests (39%), predominantly coniferous woods (29% coniferous forest, 8% broadleaf forest, and 2% mixed forest). Heaths and wetlands (swamps and peat bogs) cover about 7% of the surface.

### Discrete choice experiment

In order to analyse the multiple dimensions of rewilding as well as the trade-offs among these, a discrete choice experiment (DCE) was conducted. DCE are a survey-based approach which is particularly suited to explore preferences and willingness to pay (WTP) for changes in landscapes, here with reference to rewilding. Respondents are asked to choose between different alternatives (hypothetical scenarios), each described by attributes and attribute levels, selecting the one with the highest utility. Often DCEs include one monetary attribute in order to be able to estimate respondents’ marginal WTP for different attributes (Louviere et al. 2000). In this study, the elicitation of WTP was supplemented by a battery of follow-up questions in order to better illustrate the values and motivations regarding support for/opposition against rewilding measures.

The initial phase of the DCE design involved the identification of attributes. As previously mentioned, rewilding in a multifunctional landscape, such as the Oder Delta, covers a wide range of facets, calling for the employment of multiple approaches. Initially a comprehensive review of the literature, as well as an analysis of the current state of the landscape and of the rewilding initiatives undertaken by the transdisciplinary project partner in the case study area was conducted. As described above, only few economic studies investigated rewilding as a concept and with reference to different conceptions of it. All the rewilding concepts investigated so far differ from the more holistic approach implemented in the Oder Delta (see again Fig. 1). Given the absence of stated preference studies investigating a holistic approach to rewilding, during the survey design phase, the scope was then broadened to encompass attribute-based valuation of central aspects related to holistic rewilding, i.a., biodiversity (see, e.g., Meyerhoff et al. 2009), (changes in) landscape composition and multifunctionality (see, e.g., Bernués et al. 2019; Sagebiel et al. 2020; Immerzeel et al. 2022), peatland restoration (see, e.g., Glenk and Martin-Ortega 2018), small river restoration (Giergiczny et al. 2021), with regard to economic development (see, e.g., Foelske et al. 2019) as well as landscape aesthetics (see, e.g., Schaak and Musshoff 2020). Furthermore, the selection of attributes and their levels was informed by iterative discussions and exchanges with a range of experts involved in the project including ecologists, environmental economist, environmental political scientist, experts in sustainable development as well as local rewilding practitioners. Potential attributes were discussed in these consultations to ensure they were relevant to the ecological, socio-economic, and cultural factors of rewilding. These discussions helped ensure that the attributes reflected both scientific knowledge and local perspectives. Additionally, visits to the study area contributed to grounding attributes in both the scientific literature and practical, local insights. This resulted in the identification of six attributes to represent some of the cores of the rewilding approach in the Oder Delta described above (see Table 1 for details). Note that ‘micro-rewilding’ implies the restoration and increased connectivity of insect-friendly habitats on a farm level. Based on Rewilding Europe’s definition, rewilding enterprises were understood in terms of enterprises that generate ‘direct or indirect finance, incentives or engagement for rewilding, and has a positive impact on wilder nature or the comeback of wildlife’ (Caalders et al. 2022, p. 5). The levels of this attribute were derived from another work package within the project that identified (potential) rewilding enterprises in the case study area.

**Table 1:** Description of the attributes and relative levels as well as their links to the cores of the holistic approach to rewilding implemented in the Oder Delta

Cores of rewilding in the Oder Delta	Overarching topic	Attributes	Levels (status quo in bold)	Coding
Climate protection	Rewetting	Proportion of areas with water logging (*moorschonende Stauhaltung*)	5%, 10%, 15%	Continuous
Wildness	Naturalness	Scenery	Homogeneous, moderately heterogeneous, heterogeneous	Dummy
	Wildlife (large herbivores and carnivores)	Presence of wolf	No, yes	Dummy
		Presence of elk and European bison	No, yes	Dummy
	‘micro-rewilding’	Restoration and connectivity of insect-friendly habitats	No, yes	Dummy
Nature conservation	Human interferences and management intensity	Pesticide usage	Chemical pest control, biological pest control	Dummy
Sustainable value added	Regional development potentials	Number of local rewilding enterprises	22, 33, 44	Continuous
Costs/financing	Willingness to pay	Annual contribution to a landscape fund	0€, 10€, 25€, 50€, 80€, 110€, 160€	Continuous

Attributes were treated as dummy or continuous variables, depending on whether they were quantifiable. Continuous variables included the proportion of areas with waterlogging, the number of local rewilding enterprises and the annual contribution to a landscape fund. These were treated continuously to assess how marginal changes in these factors influenced respondents' preferences. In contrast, attributes such as the presence of wolves, elk, and European bison, restoration and connectivity of insect-friendly habitats, pesticide usage, and scenery were dummy-coded. This approach captures preferences for specific attribute levels relative to the status quo.

**Fig. 4:**
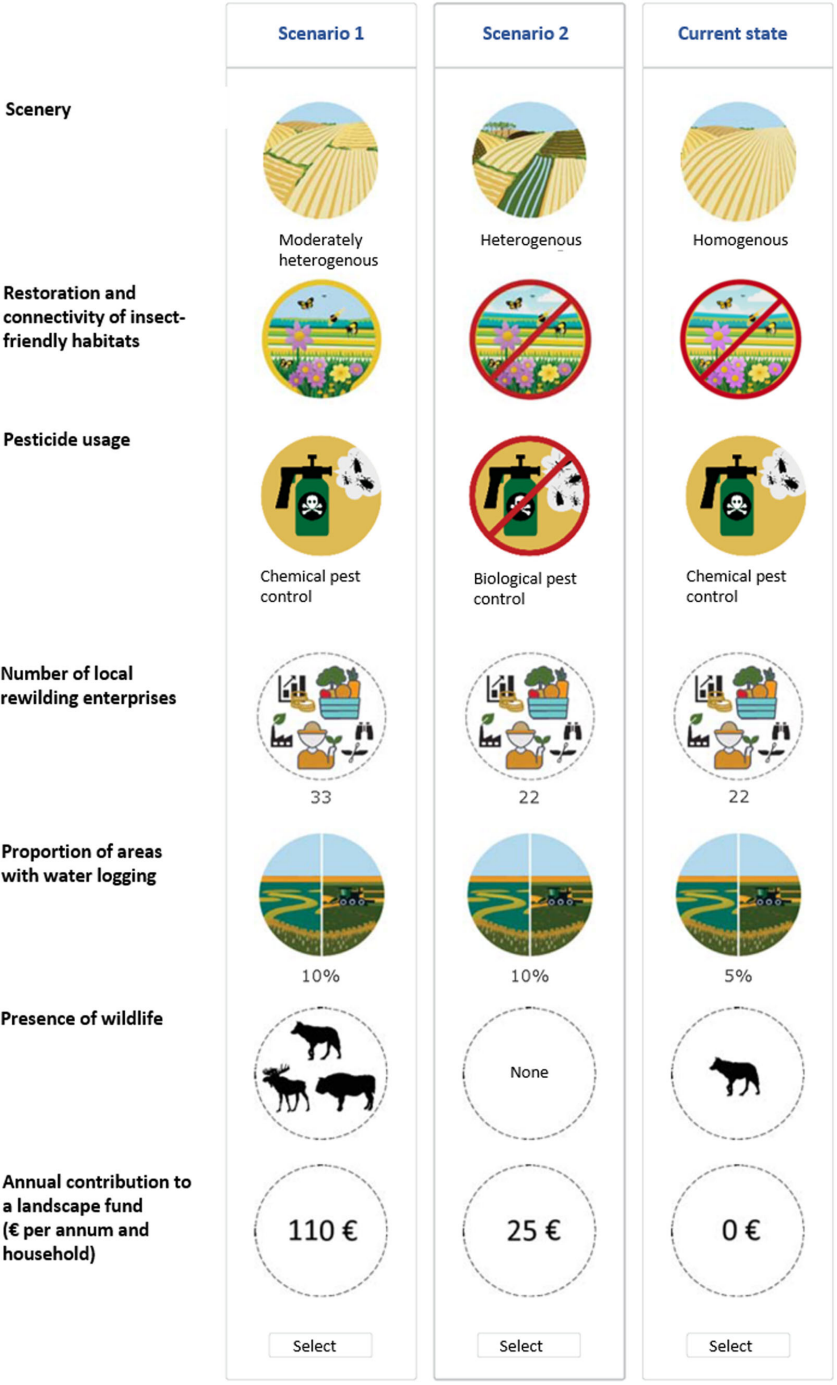
Translated example of a choice card

In the DCE the respondents were facing an unlabelled design, with two alternatives plus the status quo alternative in each choice set. Each alternative was defined by the six attributes representing a holistic approach to rewilding and a price attribute (see Fig. 4 for an example choice card). Following Sagebiel et al. (2020), who conducted a study on land use changes in Germany, an annual contribution to a newly (hypothetically) introduced landscape fund was chosen as the payment vehicle. Respondents were explained that this payment was compulsory, for a period of five years and exclusively used to finance the described changes in the naturalness of the landscape. The design of the payment vehicle as landscape fund was preferred over taxes because the rewilding measures undertaken by the transdisciplinary project partner are local and not governed by state authorities, whereas in Germany taxes are usually collected on a higher level and cannot be used for a single specific purpose only.

As it cannot be assumed that the general public is familiar with the concept of rewilding, especially not with the key natural processes (dispersal, trophic complexity and stochastic disturbances), respondents were introduced to this topic before the DCE. This was done by (i) giving an overview about the current land use in the case study area, (ii) asking about the respondents’ relation and use of the landscape, (iii) introducing and explaining the three key ecological processes of rewilding, and (iv) on this basis, introducing and explaining the attributes as well as the current status quo. At this point respondents were also asked about their satisfaction regarding the current state of the landscape in terms of the presented attributes (similar to the approach of Immerzeel et al. (2022)). Note that the term ‘rewilding’ was omitted from the survey text due to its lack of a strict definition, which introduces potential for varied connotations and could lead to uncontrolled variation, and lack of a direct translation in German. Instead, it was chosen to refer to the restoration of natural landscapes, framed in terms of three key ecological processes: dispersal, trophic complexity, and stochastic disturbances (Perino et al. 2019). Additionally, anthropogenic pressures, particularly the intensification of agriculture in the region, were introduced to provide context. Thereby, the reference to restoration of natural landscapes was aligned with the rewilding definition of Perino et al. (2019), which emphasises the restoration of these natural processes and the ongoing human pressures on landscapes.

The experimental design, created with the NGene software, consisted of 12 choice tasks and aimed to minimise the D-error. While there is no threshold what constitutes a ‘good’ D-error value, as it is case-specific and not comparable across studies, a D-error value below one suggests that the data are suitable for model estimation (Bliemer and Rose 2024). At first, a Bayesian efficient design with priors close to zero was constructed for the pre-test (*n* = 55). The design incorporated Bayesian priors (assuming that priors are random variables) to address uncertainties stemming from limited knowledge about the true parameter values (Bliemer et al. 2008). The Bayesian priors were then updated based on the pre-test results to refine the design for the main study (resulting in a D-error of 0.11).

### Analysis of motivations behind choices

In addition to the analysis of WTP, the motivations that underlie the preferences for rewilded landscapes in the Oder Delta were investigated in order to gain a better understanding about the types of preferences and values associated with the respondents’ WTP. It has been argued that transcendental values (values concerning guiding principles that transcend a given context) are often latent and have to be made explicit in the valuation process (see, e.g., Brown 1984; Niemeyer 2004; Kenter et al. 2015; Massenberg et al. 2023a). Therefore, respondents were asked to fill out a translated version of the Environmental Portrait Value Questionnaire (E-PVQ) developed by (Bouman et al. 2018) before filling out the choice cards. In the E-PVQ respondents are asked to place themselves in alignment with statements (i.e., a personal portrait) to assess biospheric, altruistic, hedonic, and egoistic values. Thereby, the E-PVQ makes transcendental values explicit and they may be considered in the valuation process (see, e.g., Massenberg et al. 2023a).

After the completion of the choice task, respondents were then asked to provide more information on the motivations underlying their choices. Table 2 provides an overview about the Likert-type items asked as follow-up questions.

**Table 2:** Overview of potential motivations behind choices asked as follow-up questions to supporters

Context		Conceptual core	Statement
Specific values	Total Economic Value	Direct use value	…because they have a personal benefit for me, for example, relaxation
		Option use value	…to preserve genetic diversity
		Non-use value—altruistic value	…so that other people can enjoy and use them
		Non-use value—bequest value	…because they should be preserved or restored for future generations
		Non-use value—existence value	…to bring plant and animal populations and the local landscape into a healthy state
		Insurance value	…because we do not know what will happen in the future
	Relational Values	Sense of place	…because they help me to feel at home
		Local identity and culture	…because it is an important part of the local culture and identity
Identified needs	Value hierarchies	Project’s legitimacy	…because the restoration of natural landscapes requires financial support
Action-outcome expectation	Perceived behaviour control	Realisability of project due to payment	…because I think that the project can be realised through the payments
We-preferences/I-preferences	Consumer citizen	Personal importance	…because it reflects the importance of natural landscapes for me
		Societal importance	…because it reflects the importance of natural landscapes for society
	Fairness	Intragenerational	…because I was thinking about what would be fair/solidary for current generations
	Deontology	Duty	…because I have a duty to pay if it serves nature conservation, biodiversity and/or sustainability
	Existence rights	Weak anthropocentric intrinsic preferences	…because plants and animals have as much rights as humans to exist
Perception of the environment and environmental attitudes	New Ecological Paradigm	Fragility of balance	…because the balance of nature is very delicate and easily upset
		Ecocrisis	…because if things continue on their present course, we will soon experience a major ecological catastrophe

The first and second columns specify the context and conceptual cores on which the statements in the last column are based (see Massenberg 2021; Massenberg et al. 2023a for details). Seventeen potentially relevant motivations behind preferences for restoration of natural landscapes were included in the analysis. These potentially relevant motivations were clustered into five overarching topics:

1. Specific values. The first set of motivational questions incorporates specific values with reference to the concepts of Total Economic Value (direct use value, option use value, altruistic value, bequest value, existence value, and insurance value) and relational values (sense of place as well as local identity and culture). Thereby, this set of motivations covers a broad range from relatively tangible values, e.g., direct use value, to intangible values such as sense of place.
2. Identified needs. This item accounts for the influence of the project’s perceived legitimacy on WTP.
3. Action-outcome expectation (the perception of the effect of an action). It is assumed that a sense of control affects respondents’ WTP (see Ajzen 1991; Spash et al. 2009). In this case, the focus lies on the perception that contributing financially to the project will actually have a positive effect in terms of an increase in the landscape’s naturalness.
4. I- and We-preferences. Drawing on the work of Vatn (2005, 2009), it is assumed that preferences may be either individually oriented (on self-interested utility maximisation) or socially oriented (associated with aspects of fairness, duty, social ends as well as consideration of interests of non-human entities).
5. Perception of the environment and environmental attitudes. Parts of the (German translation of the) New Ecological Paradigm Scale (NEP) (Dunlap et al. 2000; Schleyer-Lindenmann et al. 2018) were used in order to measure a person’s (environmental) world view, meaning beliefs and concerns about the environment. It has been shown that these influence awareness, ascription of responsibility to oneself, and personal norms (Stern 2000).

It should be noted that while each item refers to a single context and a single conceptual core, they may actually relate to several contexts as well as conceptual cores and/or be interconnected. For instance, the item on non-human entities' existence rights is linked to We-preferences because the underlying preferences are weakly anthropocentric intrinsic preferences (see Hargrove 1992; Massenberg et al. 2023a). The underlying thought is that humans may respect a non-human creature for itself, and that anthropocentrism does not always entail instrumentalism. Still, existence rights also represent one of the fifteen NEP elements, thus it could as well be a part of the NEP cluster. However, for the sake of simplicity, each statement will be linked to a single conceptual core.

Respondents who always chose the status quo were asked a different set of follow-up questions (see Table 3) in order to (i) distinguish between real zero WTP and protest, and (ii) learn more about the motivations underlying protest. As before in Table 2, the first and second columns specify the categories and conceptual cores the statements in the last column are based on. The potentially relevant motivations were clustered into three overarching topics:

1. Identified needs, preferred end states, and value indicator. This set of motivations addressed the influence of problem awareness, understood in terms of identified needs and preferred end states, on WTP. Additionally, the value indicator defined by the response mode (here: monetary) may result in protest due to incommensurability (Vatn 2009).
2. Action-outcome expectation. Also, a lack of perceived behaviour control may lead to protest choices, especially with regard to distrusting the payment vehicle or the project’s realisability to contributing to the landscape fund. Furthermore, one item in this set refers to a potential budget constraint in order to be able to distinguish real zero WTP from protest choices.
3. Perception of the environment and environmental attitudes. Also respondents who always chose the status quo option were asked two items of the (German translation of the) New Ecological Paradigm Scale (NEP) (Dunlap et al. 2000; Schleyer-Lindenmann et al. 2018) in order to understand whether their (environmental) world view bases on strong anthropocentrism as well as human exemptionalism.

**Table 3:** Overview of potential motivations behind choices asked as follow-up questions to protesters

Context		Conceptual core	Statement
Identified needs Preferred end states Value indicator	Value hierarchies	Myopic anthropocentrism	…because money should rather be used to help people in the here and now
		Project’s illegitimacy	…because the restoration of natural landscapes does not require financial support
		Commensurability	…because money is not a suitable means, a solution should not be linked to money
Action-outcome expectation	Perceived behaviour control	Budget constraint	…because I am not sure if I can afford the amount
		Distrust in payment vehicle	…because I don’t trust the type of financing
		Realisability of project due to payment	…because I do not think that the project can be realised due to the payment
Perception of the environment and environmental attitudes	New Ecological Paradigm	Anthropocentrism	…because humans have the right to modify the natural environment to suit their needs
		Human exemptionalism	…because human ingenuity will insure that we do NOT make the earth unlivable

Figures concerning the motivations were plotted in the statistical software R, using the *sjPlot* package (Lüdecke 2023).

### Data collection

The survey was web-based and respondents were recruited by a subcontracted company, Innofact AG.^1^ It was conducted in German and subsequently translated by the author. At first, a pre-test was conducted in November 2023 which consisted of a non-representative sample of 55 respondents. The two overarching aims of the pre-test were (i) to test the comprehensibility of the survey as well as relevance of the questions and attributes; and (ii) to obtain priors for the generation of the experimental design for the main survey. Based on the positive feedback of the respondents, only minor changes were made to the survey in order to increase comprehensibility. On this basis, the main survey was implemented online in December 2023. A representative sample of 350 respondents across the state of Mecklenburg–West Pomerania participated in the main survey (representativeness quotas covered gender, age, education, and location of residence).

### Econometric model

The random utility theory (McFadden 1974) provides the econometric foundation for choice experiments, which are grounded on Lancaster's consumer theory (Lancaster 1991, 1966). According to the latter, people have preferences for the qualities of goods (attributes) rather than the good itself. Therefore, it can be assumed that people base their choice on the particular characteristics of rewilding when valuing the different choice options presented in the DCE. The total utility is the sum of the utilities from all seven attributes. Thereby, it can be assumed that in the DCE respondent *n* selects the alternative *i* from a choice set *S* if this alternative derives a higher utility than the other alternatives in the choice set *j*:

$$U_{ni} > U_{nj} , \,\,\forall j \ne i \,\,\,and\,\,\, i, \,\,j \in S$$

The utility that respondent *n* derives from choosing alternative *i* in choice set *S* is defined as follows:

$$U_{ni} = V_{ni} + e_{ni} = {\ss}_{n} x_{ni} + e_{ni}$$

where *V* is the deterministic and observable utility component, *e* is the random error term capturing the unobservable influences on choice, x is the vector of the alternative’s observed characteristics (attributes), and ß is a vector of attribute coefficients as well as the alternative specific constant associated with the status quo alternative (ASC_SQ_).

Multinomial logit models (MNLs) have been fundamental for the analyse of DCEs (McFadden 1974) and still remain versatile. However, they suffer from some limitations, i.a., in terms of limited incorporation of preference heterogeneity (Bliemer and Rose 2010). Therefore, mixed logit models (MXLs) (also referred to as random parameters logit model) have become a widely applied alternative which is better able to accommodate preference heterogeneity. Thereby, covariates may be included in the analysis in order to explain variation across individuals:

$${\ss}_{nk} = {\ss}_{k} + \pi_{k} z_{n} + \sigma_{k} \varepsilon_{kn}$$

where ${\ss}_{nk}$ is the individual-specific coefficient of attribute *k* for individual *n*, ${\ss}_{k}$ is the coefficient’s constant part, $\pi_{k}$ is the vector of coefficients of individual characteristics $z_{n}$, $\sigma_{k}$ is the error term’s constant part, and $\varepsilon_{kn}$ the error term’s individual-specific component. It was assumed that all distributions followed a normal distribution apart from the monetary attribute, which was assumed to be lognormal distributed. WTP for each attribute was calculated by the following formula (Daly et al. 2012a, b; Mariel et al. 2021):

$$WTP_{k} = \widehat{{\beta_{k} }}{/}\exp \left( {\widehat{{\beta_{{{\text{price}}}} }} + \frac{{\widehat{{\sigma_{{{\text{price}}}} }}^{2} }}{2}} \right)$$

where WTP_k_ is the WTP for attribute *k,*$\widehat{{\beta_{k} }}$ is attribute’s *k* estimated parameter, $\widehat{{\beta_{{{\text{price}}}} }}$ the estimated price parameter, and $\widehat{{\sigma_{{{\text{price}}}} }}$ the estimated standard deviation of the price parameter.

To further explore preference heterogeneity and link it to motivational factors, a latent class model was estimated. The latter classifies individuals into non-predetermined segments based on similarities in preferences. The preferences for rewilded landscapes are assumed to be homogeneous within each class but vary across different classes.

The probability of individual *n* belonging to class *q* out of a set of classes Q is calculated as follows (see, e.g., Greene and Hensher 2003; Scarpa et al. 2003):

$$\Pr \left( q \right) = \frac{{\exp \left( {z_{n} \theta_{q} } \right)}}{{1 + \mathop \sum \nolimits_{j = 1}^{J - 1} \exp \left( {z_{n} \theta_{q} } \right)}}$$

where $z_{n}$ represents observable characteristics of the individual, and $\theta_{q}$ vectors of estimable parameters associated with each class. Given membership in class *q*, the probability that individual *n* selects alternative *i* from a set of *K* alternatives in choice situation *t* is represented by:

$$Pr(i|q) = \frac{{{\text{exp}}\left( {x_{nkt} \beta_{q} } \right)}}{{1 + \mathop \sum \nolimits_{k = 1}^{K} {\text{exp}}\left( {x_{nkt} \beta_{q} } \right)}}$$

where $x_{nkt}$ represents a set of observable characteristics which enter the class membership model, and $\beta_{q}$ are class-specific parameters. For a sequence of choices $T_{i}$ (in this case 12), the probability to be in class *q* is:

$$\Pr (i_{1} , \ldots i_{12} |q) = \mathop \prod \limits_{t = 1}^{t = 12} \frac{{\exp \left( {x_{nkt} \beta_{q} } \right)}}{{1 + \mathop \sum \nolimits_{k = 1}^{K} \exp \left( {x_{nkt} \beta_{q} } \right)}}$$

The log-likelihood for the model is estimated using the following formula:

$$L = \mathop \sum \limits_{i} \mathop \sum \limits_{k} I_{k} \ln \mathop \sum \limits_{c} [\Pr \left( q \right)\Pr (i_{1} , \ldots i_{12} |q)]$$

where $I_{k}$ is the indicator variable for the observed choice, and the parameters for one of the classes are normalised to zero for model identification. The inclusion of respondent-specific variables as covariates in the model helps explain how these factors influence class membership.

In this study, the variables included as covariates in the latent class model were based on respondents' motivations, as described in Table 2. To simplify the model and avoid overfitting, given the relatively small sample size, these motivational variables were grouped into five clusters (i) specific values, (ii) identified needs and action-outcome expectations, (iii) I-preferences, (iv) We-preferences, and (v) perception of the environment and environmental attitudes and transformed into dummy variables, indicating whether a respondent exhibited a strong/high motivation in each of the five clusters. All models were estimated using the statistical software R (R Core Team 2020), specifically the package ‘apollo’ (Hess and Palma 2019). In the estimation of MXL models, the resolution of simulated random parameter distributions can significantly influence the resulting solution. To ensure robustness and accuracy in the analyses, MXL models were estimated with 25,000 Sobol draws for the random distributions (Czajkowski and Budziński 2019). This approach was chosen to mitigate potential biases and uncertainties associated with parameter estimation, thereby enhancing the reliability of the findings. Standard errors were estimated using the Delta Method (Carson and Czajkowski 2019; Hess and Palma 2019).

## Results

### Descriptive statistics

The survey was completed by 350 respondents living in the state of Mecklenburg–West Pomerania, of which 37 were identified as protesters and excluded from the estimations, because they could result in inconsistent welfare estimates (see Meyerhoff and Liebe 2010).

Participants were excluded as protest votes if they chose the status quo alternative in all twelve choice sets and reported a ‘very high’ decision influence score for distrust in the finance vehicle, distrust in the project’s realisability due to contributing to the landscape fund and/or objection against money as a mean (incommensurability). The descriptive statistics of the final sample (313 respondents) are presented in Table 4. Note that following Massenberg (2021), in addition to socio-demographic characteristics, two measures considering the (self-assessed) sense of connectedness to nature and society were incorporated into the study. The Inclusion of Community in Self (ICS) scale (Mashek et al. 2006, 2007) was used to assess the connectedness to society at large, whereas the Inclusion of Nature in Self (INS) scale to measures the connectedness to nature (Schultz 2002). These scales, which use very basic pictograms, make it straightforward for survey respondents to evaluate and discuss a somewhat complex issue, like a person's relationship with nature.

**Table 4:** Descriptive statistics of respondents

Characteristic	N = 313
*Age (years)*	
Mean	46.6
Median	48.0
*Gender*	
Male	141 (45%)
Female	172 (55%)
Diverse	0 (0%)
*Residence*	
Rural	73 (23%)
Partly urban	93 (30%)
Predominantly urban	147 (47%)
*Education*	
Below Abitur	169 (54%)
Abitur or equivalent	71 (23%)
Higher education	73 (23%)
*Household monthly income*	
Below 1,000€	31 (10%)
1000–1500€	41 (13%)
1500–2000€	41 (13%)
2000–2500€	56 (18%)
2500–3500€	50 (16%)
3500–5000€	64 (21%)
Above 5000€	26 (8.4%)
Unknown	4
*Environmental organisations*	
Members	14 (4.5%)
Donated last 12 months	55 (18%)
*Connection to agriculture, forestry or fishery*	
Self or household member employed in agriculture, forestry or fishery	16 (5.1%)
Grown up on farm	20 (6.4%)
Family employed in agriculture, forestry or fishery	20 (6.4%)
Good friends employed in agriculture, forestry or fishery	48 (15%)
None	209 (67%)
*Hunting license*	25 (8.0%)
*Inclusion of Nature in Self (INS)*	
Mean	4.7
Median	5.0
*Inclusion of Community in Self (ICS)*	
Mean	3.3
Median	3.0
*High sustainable behaviour*	117 (37%)
*High willingness to sacrifice*	37 (12%)

### Estimation results

Table 5 presents the results of the estimated models. Initially, a simple MNL was estimated (Model 1). In a second step a MXL model (Model 2) incorporating only random attribute parameters was estimated in order to see whether preference heterogeneity should be accounted for. The comparison of the goodness-of-fit measures (AIC, BIC, Log-Likelihood and adjusted pseudo-*R*^2^) between Model 1 and Model 2 indicates a strong increase in the model fit. This implies that indeed a large preference heterogeneity exists within the sample. Therefore, in the following analysis and discussion of results, the focus will lie on the results of the MXL model. Additionally, to further explore preference heterogeneity a MXL model including interactions between the status quo choice and a set of individual-specific variables (with respect to socio-demographics as well as environmental attitudes and worldviews) was estimated (Model 3). However, as indicated by the slight changes in the goodness-of-fit measures, the inclusion of interactions does not seem to improve the model performance.

**Table 5:** Estimation results

	Model 1 (MNL)			Model 2 (MXL)			Model 3 (MXL with covariates)		
	Estimate	Rob. s.e	*p*-value	Estimate	Rob. s.e	*p*-value	Estimate	Rob. s.e	*p*-value
*Preference parameters*									
ASC_*SQ*_	− 11.020	1.325	0.000	− 23.663	2.306	0.000	− 22.123	2.468	0.000
Proportion of areas with water logging	0.014	0.004	0.001	0.028	0.006	0.000	0.028	0.006	0.000
Moderately heterogeneous scenery	0.230	0.048	0.000	0.430	0.070	0.000	0.426	0.070	0.000
Heterogeneous scenery	0.409	0.054	0.000	0.529	0.081	0.000	0.526	0.081	0.000
Biological pest control	0.609	0.063	0.000	0.740	0.097	0.000	0.734	0.097	0.000
Number of local rewilding enterprises	0.001	0.002	0.242	0.006	0.003	0.018	0.006	0.003	0.020
Presence of elk and European bison	0.498	0.061	0.000	1.022	0.106	0.000	1.019	0.106	0.000
Presence of wolf	0.249	0.040	0.000	0.441	0.066	0.000	0.441	0.066	0.000
Restoration and connectivity of insect-friendly habitats	0.650	0.054	0.000	1.123	0.093	0.000	1.120	0.093	0.000
Cost (€/year)	− 0.007	0.001	0.000	− 4.780	0.190	0.000	− 4.765	0.187	0.000
*Distributions of random parameters*									
sd.Proportion of areas with water logging				− 0.011	0.028	0.349	− 0.011	0.026	0.337
sd.Moderately heterogeneous scenery				0.004	0.008	0.326	0.008	0.010	0.226
sd.Heterogeneous scenery				0.198	0.192	0.150	0.165	0.245	0.250
sd. Biological pest control				1.179	0.096	0.000	1.172	0.097	0.000
sd.Number of local rewilding enterprises				− 0.011	0.010	0.132	0.010	0.013	0.226
sd.Presence of elk and European bison				0.095	0.013	0.000	0.095	0.016	0.000
sd.Presence of wolf				− 0.017	0.047	0.359	− 0.023	0.053	0.330
sd.Restoration and connectivity of insect-friendly habitats				0.634	0.101	0.000	0.627	0.101	0.000
sd.Cost (€/year)				2.174	0.219	0.000	2.110	0.258	0.000
*Interactions*									
ASC_*SQ*_:age							− 0.009	0.013	0.248
ASC_*SQ*_:gender							− 0.290	0.348	0.203
ASC_*SQ*_:countryside							− 1.004	0.361	0.003
ASC_*SQ*_:higher_education							− 0.906	0.415	0.014
ASC_*SQ*_:donation							− 0.559	0.427	0.095
ASC_*SQ*_:no_fff							− 0.027	0.354	0.470
ASC_*SQ*_:high_INS							0.060	0.374	0.436
ASC_*SQ*_:sust.behav							− 0.152	0.388	0.347
ASC_*SQ*_:wts							− 0.265	0.564	0.319
*Model statistics*									
N (observations)	3756			3756			3756		
N (individuals)	313			313			313		
AIC	7319.23			5549.57			5551.55		
BIC	7381.54			5667.96			5726.02		
Log-likelihood	− 3649.61			− 2755.79			− 2747.77		
Adj. Pseudo-R^2^	0.1021			0.3193			0.3191		

The first variable *ASC*_*SQ*_ represents the alternative specific constant for the status quo. The ASC_*SQ*_ for the status quo captures the average effect of all unobserved factors that influence the utility of the status quo relative to the other alternatives. The ASC_*SQ*_ is negative and significant, indicating a strong preference against the status quo option. This suggests that respondents were generally inclined to choose one of the alternative options presented to them. Put differently, on average respondents preferred to move away from the current situation and shift to one of the alternative options. The relatively large size of the ASC_*SQ*_ may reflect dissatisfaction with the current situation, particularly in the context of environmental or land management practices, and a general interest in change, representing the opposite of a typical status quo bias (see, e.g., Schaafsma et al. 2019).

Regarding the preference parameters, all attributes are highly significant in the MXL model without interactions (Model 2). The coefficient for the proportion of areas with water logging was positive, suggesting that respondents had a favourable view of rewetting some of the area. Similarly, the coefficients for more natural scenery (*moderately heterogeneous* and *heterogeneous*) were positive, indicating that respondents valued a more natural and diversified landscape. Furthermore, participants expressed strong preferences for biological pest control, as indicated by the associated coefficient *pesticide usage*. Despite being positive and significant, the estimator for the number of rewilding enterprises is close to zero, which shows that the respondents cared more about other aspects of rewilded landscapes whereas new business opportunities by rewilding enterprises appear less relevant. While the participants expressed strong preferences for the presence of large herbivores (*presence of elk and European bison*), the wolf seems to be perceived as negatively. Note that wolves are already present in the case study area and therefore the status quo was defined by their presence whereas the alternative was their disappearance. Thus, the positive and significant estimator *presence of wolf* in this case indicates that people prefer the absence of wolves in the case study area. In contrast, the restoration and increase in connectivity of insect-friendly habitats was highly valued by the participants. As expected, the cost coefficient was negative and statistically significant, suggesting that respondents were sensitive to the cost of the alternatives and preferred lower-cost options.

Regarding the distributions of random parameters, the standard deviations revealed significant heterogeneity in preferences for some but not all attributes. There was significant variability in how respondents viewed pest control, presence of large herbivores, insect-friendly habitats, and cost. This suggests that respondents had varying preferences for these attributes, with some individuals valuing them more highly than others. In contrast, the results do not indicate a significant heterogeneity in preferences for the proportion of areas with water logging, landscape diversity, rewilding enterprises and wolf presence, indicating that respondents had more consistent preferences for these attributes.

The third model, the MXL model which incorporates interactions with individual-specific variables in order to explain heterogeneity in the status quo choices, shows overall similar results to the MXL model without interactions. Regarding the individual-specific variables that may affect to choose the status quo alternative, only two of them were found to be significant, indicating that participants living in the countryside and participants with higher education were less likely to choose the status quo. On the contrary, age, gender, having donated within the last twelve months to an environmental organisation (*donation*), no connection to agriculture, forestry or fishery (*no_fff*), a (subjectively) high connectedness to nature (*high_INS*), self-reported high sustainable behaviour (*sust.behav*), as well as self-reported high willingness to sacrifice (*wts*) did not seem to affect preferences for the status quo.

### WTP results

Table 6 reports the WTP values for the various attributes which vary to a large extent. Participants were willing to pay most for ‘micro-rewilding’—efforts to restore and connect insect-friendly habitats—followed by the presence of elk and European bison. Also, a shift from chemical pesticides towards biological pest control is generally valued higher by the general public relative to some of the other attributes.

**Table 6:** WTP estimates (€ per annum) for rewilding in the Oder Delta

	Model 2—Mixed logit in preference space (Delta method)	
	Mean	Standard deviation
Proportion of areas with water logging (%)	0.31	0.15
Moderate heterogeneous scenery (homogeneous is the reference level)	4.83	2.08
Heterogeneous scenery (homogeneous is the reference level)	5.94	2.55
Biological pest control (chemical pest control is the reference level)	8.30	3.49
Number of local rewilding enterprises	0.07	0.04
Presence of elk and European bison (not present is the reference level)	11.46	4.77
Presence of wolf (present is the reference level)	4.95	2.15
Restoration and connectivity of insect-friendly habitats (no efforts is the reference level)	12.60	5.26

Interestingly, participants showed only minor sensitivity to the degree of improvement in landscape naturalness (scenery) as the WTP for a shift from homogeneous (base level) to moderately heterogeneous is much higher than for the corresponding shift from moderately homogeneous to heterogeneous. The WTP for rewetting and especially an increase in local rewilding enterprises is relatively low. As discussed above, the WTP for the presence of wolves can even be considered negative. WTP for the best hypothetical scenario from a rewilding perspective (highest proportion of areas with waterlogging, heterogeneous scenery, biological pest control, highest number of local rewilding enterprises, presence of large herbivores as well as wolves, and ‘micro-rewilding’) is €42.90 per year.

### Preference and motivational heterogeneity

**Fig. 5:**
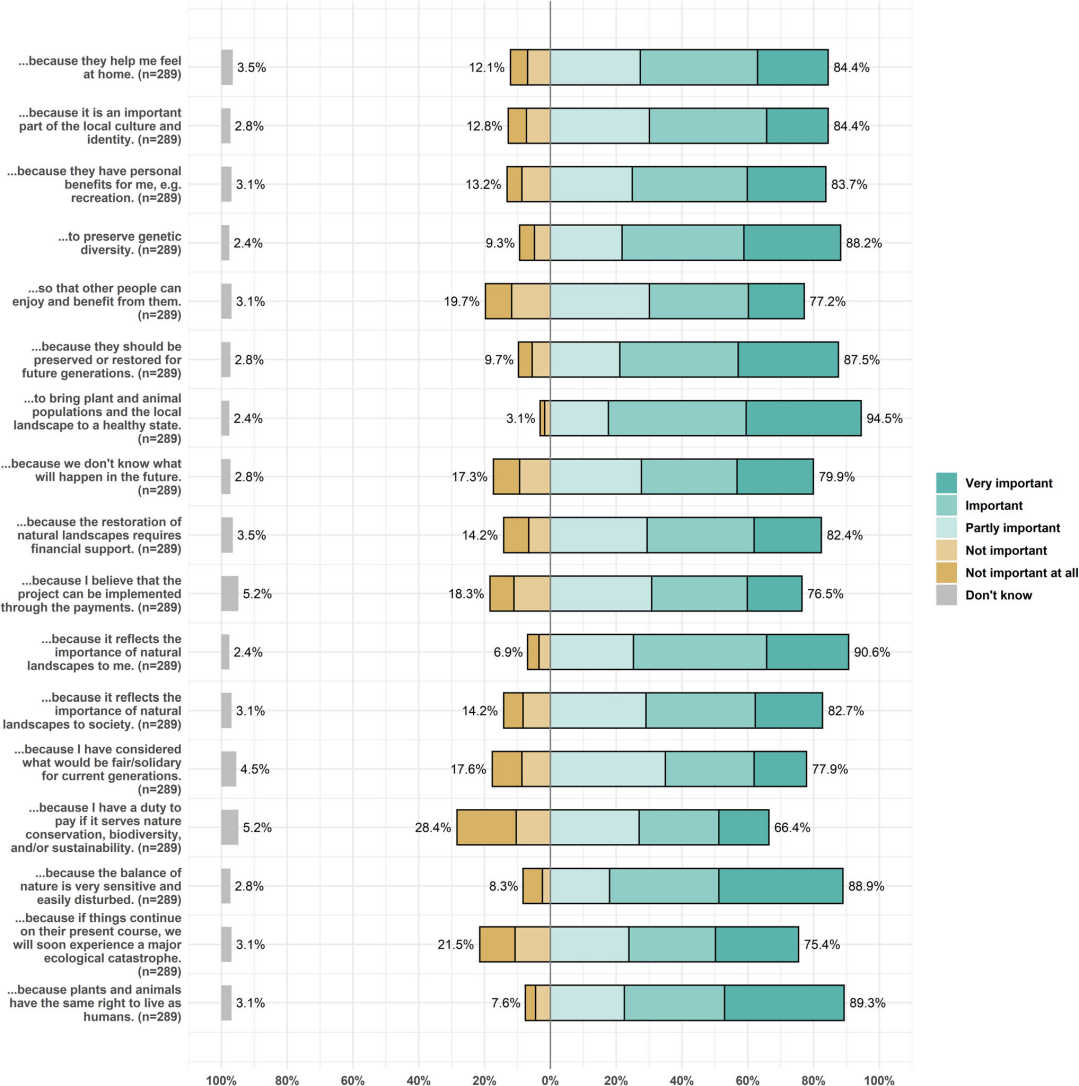
Results on underlying motivations behind stated preferences

The analysis of participants' responses to the follow-up questions reveals diverse motivations underlying preferences for natural landscapes. Across the spectrum of theories and concepts explored, all motivations were considered at least partly important by a majority of the sample (Fig. 5).

In alignment with specific values, participants considered a range of motivations referring to the total economic value (TEV) framework as important. Out of the six motivation categories associated with the TEV, altruistic value was considered the least important. Still, 77% of participants considered altruistic value at least partly important. Also, only slightly more participants (79%) were motivated by aspects of insurance value. The most important motivation, both for the TEV subset and overall, was existence value (94% of participants considered it at least partly important for their choices). Direct use value, option value, and bequest value were also stated to be important motivations (for 84%, 88%, and 87% of participants at least partly important). In this line, also relational values were considered as relevant by 84% of participants both in terms of sense of place and local culture and identity, highlighting the significance of social relationships and emotional connections in shaping landscape preferences.

Although, the protesters are not included in this overview, only 82% of respondents identified a need to financially support the restoration of natural landscapes. Also, the perceived behaviour control was a less relevant motivation relative to the others, around 77% believed that their financial contribution would lead to the realisation of the described measures.

With respect to We-preferences and I-preferences, participants expressed that on average the personal importance was more important than the societal importance (91% versus 83%). Furthermore, twice as many participants didn’t consider the societal importance as motivation compared to the individual importance (14% versus 7%). Thus, it appears that participants neither expressed pure consumer preferences nor pure citizen preferences but that many participants considered both aspects as relevant for their choices. In this regard also the importance of existence rights of plants and animals (89%), intergenerational fairness (78%) and even a felt duty to support the cause (66%) were considered as relevant by many participants. This is in line with the overall perception of the environment and associated attitudes, as 89% of participants state that they were motivated by the fragility of natures’ balance and 75% by the thought of a major ecological catastrophe if things continue as of now.

**Fig. 6:**
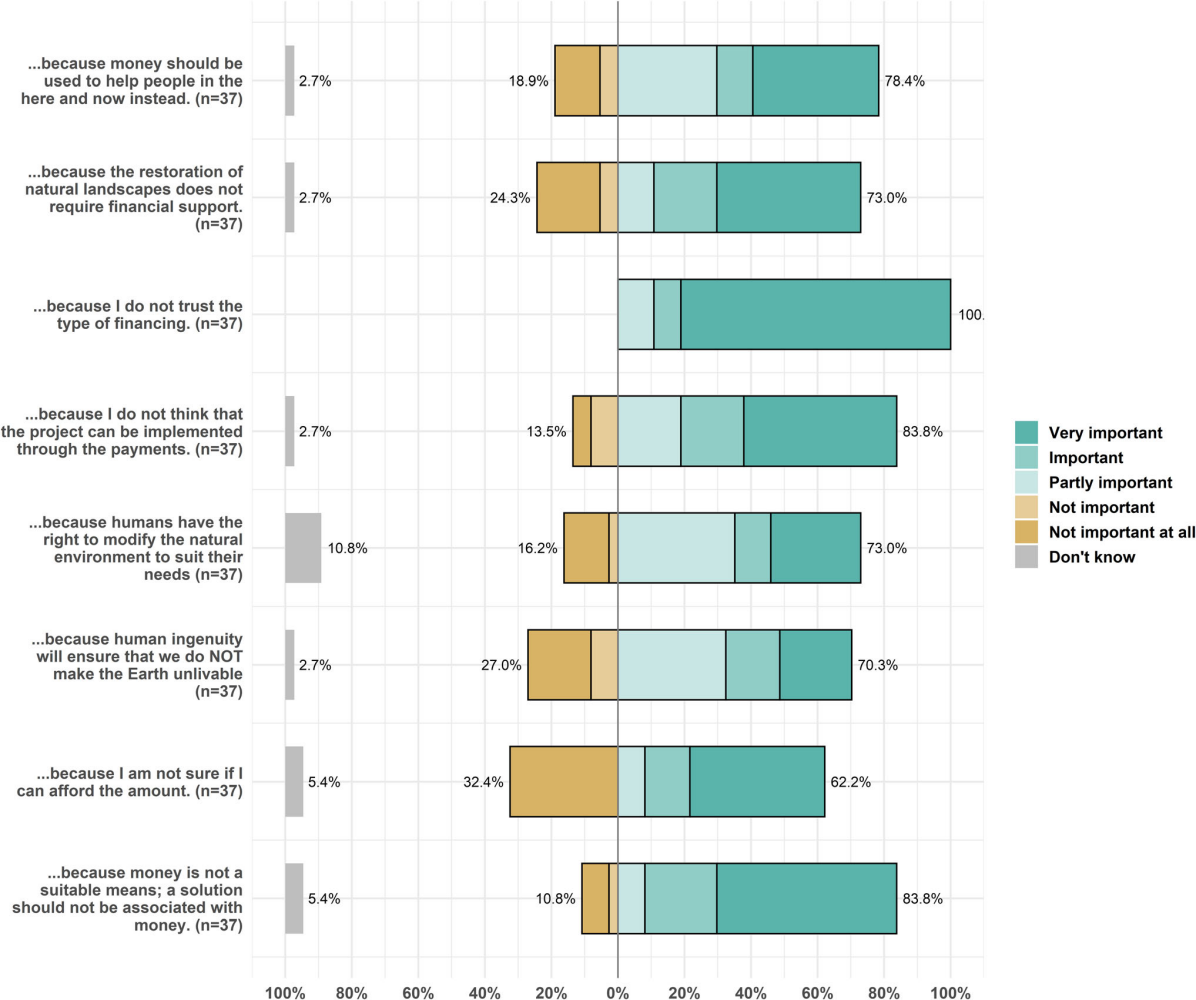
Results on underlying motivations behind protest

Turning to the underlying motivations of the protesters, these appear to be equally multifaceted (Fig. 6). Although, the biggest concern was related to distrusting the financing vehicle which all the protesters stated at least as partly important for their choices. In terms of the other motivations related to perceived behaviour control, also the distrust in the realisability of the project to a financial contribution played an important role (84% of participants thought of it as at least partly important). On the other hand, also 62% were expressing a budget constraint to some extent. With respect to value hierarchies 84% of protesters regarded (in-)commensurability as at least partly important, 73% questioned the legitimacy of the financial need to support the restoration of natural landscapes and a little more (78%) expressed myopic anthropocentrism as motivator. Again, this is in line with the overall perception of the environment and associated attitudes, as 73% stated that the human right to modify natural environments to their needs motivated them at least to some degree, and 70% believe in human exemptionalism.

To further investigate the heterogeneity in respondents' preferences revealed by the MXL model, a latent class model with three classes was employed, incorporating the motivations identified through the Likert-scale responses (see Table 7). The results show differences in magnitude, parameter signs, and statistical significance, indicating distinct preference patterns across the classes. In the following, the preference patterns and the effect of the motives on class membership will be explored. Statistically significant coefficients are interpreted relative to the probability of belonging to the reference group (class 1). Specifically, a positive coefficient suggests an increased likelihood of belonging to class 2 or class 3 compared to class 1, while a negative coefficient indicates a decreased likelihood of belonging to these classes.

**Table 7:** Regression results for latent class model with three classes

	Class 1			Class 2			Class 3		
	Estimate	Rob. s.e	*p*-value	Estimate	Rob. s.e	*p*-value	Estimate	Rob. s.e	*p*-value
*Preference parameters*									
ASC_*SQ*_	− 6.715	3.480	0.027	2.456	8.236	0.383	− 28.333	3.881	0.000
Proportion of areas with water logging	0.019	0.011	0.037	− 0.136	0.076	0.036	0.042	0.010	0.000
Moderately heterogeneous scenery	0.506	0.124	0.000	− 1.419	0.513	0.003	0.490	0.093	0.000
Heterogeneous scenery	0.804	0.127	0.000	− 0.471	0.452	0.149	0.530	0.112	0.000
Biological pest control	0.190	0.133	0.078	0.356	0.343	0.150	0.775	0.120	0.000
Number of local rewilding enterprises	− 0.009	0.005	0.037	− 0.042	0.029	0.074	0.014	0.004	0.000
Presence of elk and European bison	0.250	0.160	0.059	− 0.113	0.345	0.371	1.269	0.188	0.000
Presence of wolf	0.155	0.106	0.071	− 0.026	0.355	0.471	0.534	0.092	0.000
Restoration and connectivity of insect-friendly habitats	0.757	0.169	0.000	− 0.667	0.299	0.013	1.175	0.121	0.000
Cost (€/year)	− 0.021	0.003	0.000	− 0.012	0.007	0.030	− 0.004	0.001	0.000
*Class membership parameters*									
Intercept				− 0.012	0.280	0.483	− 0.390	0.282	0.083
Strong specific values				− 0.524	0.780	0.251	0.666	0.509	0.096
Strong identified needs and action-outcome expectation				− 0.727	0.529	0.085	0.101	0.389	0.397
Strong I-preferences				− 1.435	0.518	0.003	0.514	0.349	0.070
Strong We-preferences				1.428	0.709	0.022	0.215	0.562	0.351
Strong perception of the environment and environmental attitudes				− 1.238	0.518	0.008	0.705	0.374	0.030
*Class shares*	0.299			0.141			0.560		
*Model statistics*									
N (observations)	3756								
N (individuals)	313								
AIC	5716.42								
BIC	5978.13								
Log-likelihood	− 2816.21								
Adj. Pseudo-R^2^	0.2998								

Class 1 is predicted to represent 29.9% of the sample. Individuals in this class generally support rewilding measures but not necessarily all of them and not to the same degree. While the preferences for attributes such as proportion of areas with water logging, moderately heterogeneous and heterogeneous scenery, as well as restoration and connectivity of insect-friendly habitats are positive and highly significant, biological pest control and presence of herbivores are only significant at a 10% level. In contrast, the number of local rewilding enterprises is perceived negatively and so are wolves (although the latter again only at a 10% significance level).

Class 2 has the lowest predicted share (14.1% of the individuals). In general, individuals in this class favour the status quo over rewilding as illustrated by the positive and statistically significant ASC_*SQ*_ parameter as well as the fact that all significant attributes have a negative sign. Thus, the statistical significance of proportion of areas with water logging, moderately heterogeneous scenery, and restoration and connectivity of insect-friendly habitats indicates an opposition against these aspects of rewilding. Also rewilding enterprises are perceived negatively, although this attribute is only significant at the 10% level. Membership in this class is influenced by several motivational constructs. Surprisingly, individuals in class 2 are less likely to have strong I-preferences and more likely to be motivated by strong We-preferences. Furthermore, they are less likely driven by environmental attitudes (less concerned about fragility of balance and ecocrisis). The negative sign of the latent variable of strong identified needs and action-outcome expectation suggests that individuals in this class are less likely to see a need for action and have less perceived behaviour control (although this latent variable is only significant at the 10% level).

Class 3 comprises the majority of the sample, with 56.0% of individuals predicted to belong to this class. It is the only class that has statistically significant preferences for all attributes. Furthermore, this class has a very high ASC_*SQ*_, highlighting a strong overall support for rewilding. One explanation may be the high relevance of environmental attitudes compared to the other two classes. Thus, members of this class may not only be considering specific rewilding efforts but may also be motivated by a general concern about the state of the natural world and the risk of an ecocrisis. Furthermore, compared to the other classes members of class 3 are more likely to be motivated by strong specific values as well as I-preferences, although both latent variables are only significant at a 10% level.

## Discussion

In this study, particularly, the preferences for increasing wildness exhibit multifaceted patterns. While respondents expressed high WTP for enhancing wildness through micro-rewilding and the presence of large herbivores, the valuation of a more heterogeneous landscape showed a nonlinear trend. Interestingly, the shift from a homogeneous status quo to moderately heterogeneous was valued higher than the corresponding shift from moderately heterogeneous heterogeneous. However, the presence of wolves, was perceived negatively by respondents, highlighting that not every increase in wildness is perceived positively. A potential for conflicts arises as wolves are habitat generalist which can adopt to all kind of habitats (Fechter and Storch 2014). Particularly, in the case of species conservation it has been shown before that a certain species may be perceived as public good by some and as public bad by others (Bostedt 1999). Furthermore, the social and cultural context affects attitudes towards wolves including cultural-specific stereotypes (Kleiven et al. 2004; Jürgens and Hackett 2017). Thus, the preferences observed in this study may stem from the negative reputation of wolves in Germany, rooted in history and cultural representation, indicating a need for more extensive environmental education. Moreover, preferences for reduced human interference and management intensity were observed, aligning with the highly perceived relevance of associated values and motivations, such as concerns about disturbing the natural balance, risks of an ecocrisis, and recognition of existence rights and value of non-human entities. These findings underscore the intricate interplay between ecological, cultural, and societal factors shaping public preferences and attitudes towards rewilding initiatives in the Oder Delta. Overall, the findings regarding ecological aspects of rewilding are consistent with a recent study by Dunn-Capper et al. (2024), which investigated WTP for the ecological concept of rewilding developed by Perino et al. (2019). Their study assessed public preferences on a German national level for rewilding interventions in the Oder Delta compared to an intensification scenario. The authors show that the general public is willing to pay for various rewilding attributes, but that on a more regional level aspects are also perceived negatively, such as the presence of wolves.

Furthermore, it was found that the regional development potentials were not considered important by the participants, raising questions about the factors driving their perception. One possible explanation could be that nature-based tourism holds significant potential for wildlife-based tourism and wildlife-based economic growth in the case study area. Yet, the dominance of tourism in the coastal areas of the case study and associated concerns about over-tourism may have overshadowed the perceived benefits of regional development.

Also the protesters articulated multifaceted reasons for their opposition, reflecting a complex interplay of factors. These included a fundamental distrust in the payment vehicle associated with rewilding initiatives, as well as a more anthropocentric worldview. Additionally, there was a tendency among protesters to express scepticism about the potential consequences of their choices. Thus, the findings support existing literature that illustrated how willingness to pay for ecosystem services is influenced by perceived behavioural control (Liebe et al. 2011), as well as normative beliefs (Franceschinis et al. 2022).

While it could be valuable to explore the relationship between socio-demographic factors and preferences towards rewilding, the analysis did not reveal significant findings in this regard. The MXL model, which included interactions with individual-specific variables, showed overall similar results to the model without interactions. Specifically, only two socio-demographic variables—living in the countryside and higher education—were found to significantly reduce the likelihood of choosing the status quo. This suggests that preferences for or against rewilding, at least in this study, might be driven to a larger extent by other factors, such as attitudes towards nature and transcendental values, rather than socio-demographic characteristics.

In examining the underlying motivations of respondents, the study revealed expressions of non-individualistic, non-instrumental, and/or weakly anthropocentric considerations. Notably, it was found that a high perceived personal importance was expressed simultaneously with a high perceived importance for society. Respondents appeared to adopt both consumer and citizen perspectives, indicating a nuanced approach to evaluating rewilding. This observation challenges the traditional dichotomy between consumer and citizen perspectives (Sagoff 1998; Vatn 2005, 2009; Massenberg et al. 2023a), suggesting that individual-oriented as well as community-oriented motivations for paying for rewilded landscapes may be complementary rather than mutually exclusive. By expanding the set of considered motivations to encompass less individualistic, utilitarian, and anthropocentric considerations, this study sheds additional light on the motivational complexity and heterogeneity underlying preferences for environmental public goods. The latent class model confirmed the presence of preference heterogeneity, with different segments of respondents holding diverging views on the importance of rewilding in general as well as on specific attributes. While the motivations explored through the follow-up questions revealed diverse underlying factors influencing preferences for rewilding, the latent class model provided a clearer picture of how these motivations translate into actual preferences and how these motivations affect the likelihood of being a member of one of the segments. These findings underscore the importance of recognising and incorporating diverse motivational factors in understanding public attitudes towards environmental conservation and restoration efforts (see also Massenberg et al. 2023a).

As discussed above, overall the findings of this study indicate public support for rewilding initiatives in the Oder Delta, which could contribute to the goals of the EU Biodiversity Strategy for 2030, such as with respect to restoration of damaged ecosystems, creation of landscape elements with high biodiversity, reduction of use of pesticides, promotion of agroecological practices, and reversing the decline of pollinators. While the financial resources made available for nature conservation by the Biodiversity Strategy are substantial, they remain limited. Against this background, understanding which aspects of rewilding the general public is most willing to support can help prioritise funding, ensuring that efforts are directed where they are likely to be most accepted.

Thereby, policymakers could be supported in identifying scenarios that are most supported or least supported by the general public and rewilding initiatives may determine the feasibility of securing additional monetary support. However, the findings of this study also revealed significant variability in respondents’ opinions for some attributes (pest control, presence of large herbivores, insect-friendly habitats), whereas for other attributes (including proportion of areas with water logging, landscape diversity, rewilding enterprises and wolf presence) no or less heterogeneity in preferences was found. This finding suggests that policies targeting at attributes with more consistent preferences (non-heterogeneousattributes) might be easier to design and implement, with more predictable public support and local acceptance. Conversely, attributes with heterogeneous preferences may require tailored approaches to address the varying opinions. Inappropriately addressing this heterogeneous perception could lead to reduced support and lower acceptance. As the public opinion on these attributes appears to be more divided, policymakers should be mindful of this variability when designing policies.

Acknowledging several limitations of this study is imperative, as they also highlight significant research gaps that require attention. Future research should aim to incorporate the inherent uncertainty associated with rewilding. This uncertainty arises from the fact that rewilding processes are not entirely human-controlled and can yield unpredictable ecological outcomes and result in novel ecosystems, complicating the assessment of their economic value. Moreover, the long-term and indirect effects of rewilding on ecosystem services present challenges in economic valuation. For instance, the impact of rewilding on biodiversity and subsequent benefits to nature-based tourism or natural resource availability may unfold over decades, posing difficulties in capturing these future benefits within present valuations which elicit public preferences at one moment in time. Addressing these challenges requires repeated assessments of (changes in) public preferences as well as innovative approaches. For example, Mavrommati et al. (2017) applied a deliberative multicriteria evaluation method which asks respondents to serve as trustees for future generations instead of considering their current preferences. Thereby, the authors argue that the considered time frame is more appropriate for sustainability-related research questions. Additionally, it is crucial to account for uncertainty and variability in ecosystem service provision, given the dynamic nature of ecosystems and their susceptibility to various environmental and socio-economic factors. Thus, assessing the economic value of rewilding necessitates accounting for these uncertainties and variability in order to provide a more accurate representation of associated values and potential economic benefits. This underscores the importance of understanding the diverse motivations and attitudes shaping public perceptions of rewilding initiatives and highlights the need for nuanced strategies to address stakeholder concerns.

## Conclusions

This study aimed to address a significant research gap by examining public preferences and underlying motivations for a holistic rewilding approach, recognising the potential of economic valuation to inform policy, planning, and management decisions regarding public environmental goods. As mentioned earlier, the concept of rewilding is fuzzy which poses challenges for its economic valuation. Economic studies may focus on entirely different aspects of rewilding based on their reference approach. Thereby, some studies might evaluate rewilding only in terms of ecological conditions and effects, while others might adopt a more holistic perspective, incorporating socio-economic and socio-cultural aspects. Yet, the more complex the rewilding approach under investigation is, the more challenging its valuation becomes. The multidimensionality of (holistic) rewilding complicates the design of choice experiments, as including too many attributes can overwhelm the participants. Additionally, comparing results between studies becomes difficult if different rewilding approaches are referred to, further complicating the comparison and synthesis of findings.

The findings of this study demonstrate the existence of public preferences and willingness to pay for a holistic rewilding in the Oder Delta, suggesting that successful rewilding initiatives may contribute to key goals outlined in the EU Biodiversity Strategy for 2030. However, the multidimensional nature of public preferences and respective willingness to pay, alongside underlying motivations, underscores the complexity inherent in navigating trade-offs and conflicts arising from heterogeneous preferences and motivations, as noted in previous research (Cavender-Bares et al. 2015; Daw et al. 2015). Notably, different stakeholder groups, such as hunters and wildlife watchers, hold divergent norms (see, e.g., Ruddell and Gramann 1994), values (see, e.g., Saremba and Gill 1991), and/or beliefs (Carothers et al. 2001), which may lead to conflicts if not addressed. Therefore, fostering citizens' acceptance of rewilding is crucial, necessitating mutual understanding and engagement.

Moreover, policy decisions regarding rewilding must consider the "not-in-my-backyard" (NIMBY) phenomenon. For example, the study by Dunn-Capper et al. (2024) found that NIMBY attitudes can significantly influence local acceptance of rewilding projects, particularly concerning the reintroduction of carnivores. Although, it should be noted that the authors defined "local" relatively broadly as within 100 km of the rewilding area and in a rural setting. As mentioned earlier, this aligns with the findings of this study, whereas the overall sentiment towards carnivores in Germany is generally positive (Forsa 2024). Thus, local opposition can pose substantial challenges to rewilding efforts.

To address the challenge of (local) opposition, environmental education can play a role, but inclusive processes that involve stakeholders may be even more critical. Co-creation, combining local knowledge with scientific expertise, may support the upscaling of successful rewilding practices. Transparency in information dissemination and decision-making, alongside opportunities for civic engagement, are essential for building trust and legitimacy. Overall, this highlights the need for rewilding policies that effectively balance local concerns with broader ecological and societal benefits.

Further research is needed to gain a better understanding of the determinants of preference and willingness to pay, particularly how these evolve through participative processes, and to explore how different psychographic and value profiles influence perceptions of and preferences for rewilding. This research should be context-specific, emphasising the importance of evidence from diverse rewilding sites across Europe to provide comprehensive insights into the complexities of public preferences and attitudes towards rewilding initiatives.

## Data Availability

The data that support the findings of this study are available upon reasonable request from the author.

## Footnotes

1 https://innofact-marktforschung.de/
